# Supplementary material for: Multi-scale spatio-temporal analysis of human mobility
Source: PLoS One. 2017 Feb 15;12(2):e0171686. doi: 10.1371/journal.pone.0171686 (PMC5310761; doi:10.1371/journal.pone.0171686)
Supplement: S1 File — (PDF) [file pone.0171686.s001.pdf]

Multi-Scale Spatio-Temporal Analysis of Human Mobility Supporting Information

Laura Alessandretti[1], Piotr Sapiezynski[2], Sune Lehmann[2,3], Andrea Baronchelli[1,*]

1 City, University of London, London EC1V 0HB, United Kingdom.
2 Technical University of Denmark, DK-2800 Kgs. Lyngby, Denmark.
3 Niels Bohr Institute, University of Copenhagen, DK-2100 København Ø, Denmark.

*Email: Andrea.Baronchelli.1@city.ac.uk

1 Data pre-processing

Determining routers' locations

We determine the routers' locations using the approach described in [1] with a slight modification. The original method used only GPS location estimations calculated at the same second as a corresponding WiFi scan. Here, we consider all location estimations from Android Location API, including network based estimations. Additionally, we relax the same-second requirement as follows. In the spatio-temporal trace of each user we identify periods from time t_0 to time t_N where the user was stationary, also referred to as stop locations. This means that the distance between the user's location at t_0 and t_N is below d meters, and that there exist a location estimation between t_0 and t_N at least every n seconds. Also, it implies that each location estimation within the stop location is within d from the user's location at t_N. At t_N, the individual stop-location changes. We select n as 305 seconds, thus requiring no missing data, since the sampling period of GPS location in the experiment is approximately 300 seconds. We select d as 30 meters, a safe range compared to the typical GPS errors, thus requiring that the user remains in the same location within the resolution of a building. After identifying these stop locations, we assign the geometric median position of estimations to all routers scanned in these periods.

2 Robustness of results

Results of the model selection

The selection of the log-normal distribution as the best model among the exponential, the log-normal and the Pareto distribution is made using the Akaike Information Criterion (AIC) weights. In tables S1,S2,S3 we report the AIC weights values for the four models considered as well as the Akaike information Criterion (AIC), the Bayesian Information Criterion (BIC) weights, the Residual Sum of Squares (RSS). These metrics provide additional information on the goodness-of-fit. In figures S1, S2, S3, we show the results of the fit with the three distributions considered.

Bootstrapping

By bootstrapping data 1000 times for samples of 100 and 200 individuals, we find that for all groups the aggregated distributions of displacements and waiting times are best described by the same models found for the entire dataset.

	AIC	AIC weights	BIC weights	RSS
expon	2.1e+07	0	0	3.1e-11
lognorm	1.9e+07	1	1	2.9e-11
pareto	2.0e+07	0	0	2.8e-11

Table S1. Distribution of displacements: model selection. For the three distributions considered, the table reports the Akaike Information Criterion (AIC), the AIC weights (see Model selection section), the Bayesian Information Criterion (BIC) and the residual sum of squares (RSS).

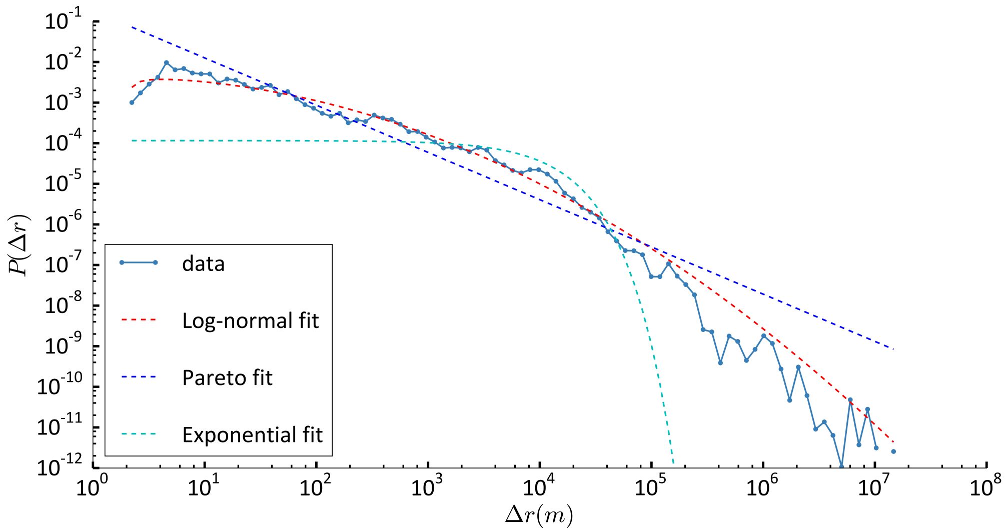

Fig S1. Distribution of displacements: comparison of three models. Blue dotted line: data. Red dashed line: Maximum likelihood Log-normal fit. Blue dashed line: Maximum likelihood Pareto fit. Light blue dashed line: Maximum likelihood Exponential fit.

	AIC	AIC weights	BIC weights	RSS
expon	4.62e+06	0	0	0.061
lognorm	3.68e+06	1	1	0.026
pareto	3.79e+06	0	0	0.025

Table S2. Distribution of waiting times: model selection. For the three distributions considered, the table reports the Akaike Information Criterion (AIC), the AIC weights (see Model selection section), the Bayesian Information Criterion (BIC) and the residual sum of squares (RSS).

Here, we report the distribution of parameters found for the distribution of displacements (Fig S4), waiting times (Fig S5), and displacements between discoveries (Fig S6), in the case of samples of 100 individuals.

Sensitivity to the definition of pausing

The distribution of displacements is robust with respect to the definition of *pausing*. The results reported in the main text refer to pauses longer than $P = 10$ minutes. Both for

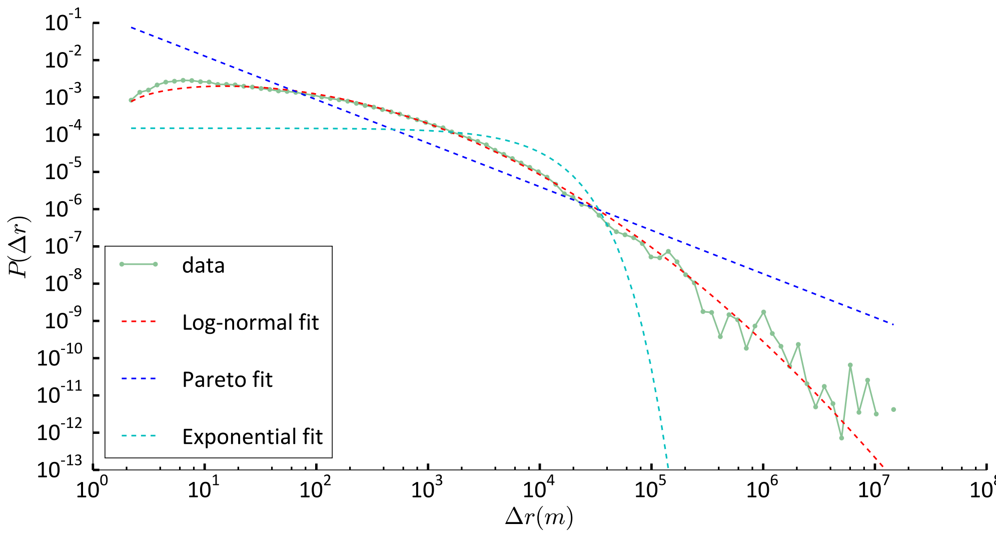

Fig S2. Distribution of waiting times: comparison of three models. Yellow dotted line: data. Red dashed line: Maximum likelihood Log-normal fit. Blue dashed line: Maximum likelihood Pareto fit. Light blue dashed line: Maximum likelihood Exponential fit.

	AIC	AIC weights	BIC weights	RSS
lognorm	2.7e+07	1	1	3.0e-11
pareto	2.9e+07	0	0	2.8e-11
expon	3.0e+07	0	0	3.1e-11

Table S3. Distribution of displacements between discoveries: model selection. For the three distributions considered, the table reports the Akaike Information Criterion (AIC), the AIC weights (see Model selection section), the Bayesian Information Criterion (BIC) and the residual sum of squares (RSS).

$P = 15$ minutes and $P = 20$ minutes, the distribution of displacements is best described by a log-normal model when the entire distribution is taken into account, and by a Pareto distribution, when only long distances are considered (see Figures S7 and S8). The same results hold for the distributions of waiting times (see Figures S9 and S10)

Interpretation of the shift and scale parameters

The shift and scale parameters are necessary to account for the fact that, in the cases considered, the lower bound of the distributions support is controlled by the data minimal resolution.

For example, the log-normal distribution of a random variable x is defined for $x \in (0, \infty)$. In our case the fit is performed for a shifted distribution, with $x \in (x_0, \infty)$, where x_0 is the data minimal resolution. This reflects the fact that the reason why there are no data points for $x < x_0$ is not low probability but lack of information within this range (or in some cases it's due to the choice of fitting only the tail of the distribution).

Similarly, the Pareto distribution is defined for $x \in (1, \infty)$. The shift x_0 and the scale parameter s allow instead to consider $x \in (s + x_0, \infty)$, where $s + x_0$ is the minimum data point considered. Values of the shift and scale parameters could be set to fit the minimal resolution. However, in our case x_0 and s are additional parameters

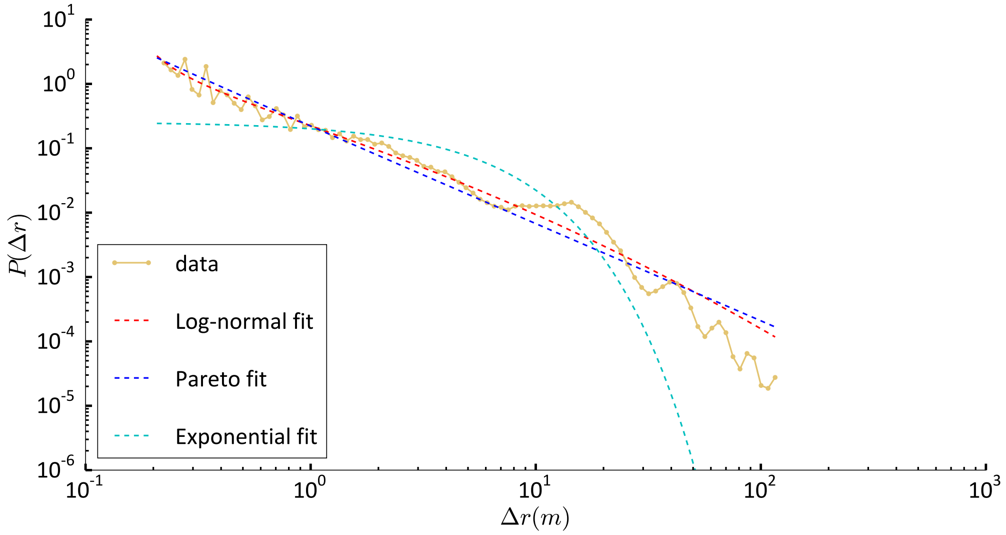

Fig S3. Distribution of displacements between discoveries: comparison of three models. Green dotted line: data. Red dashed line: Maximum likelihood Log-normal fit. Blue dashed line: Maximum likelihood Pareto fit. Light blue dashed line: Maximum likelihood Exponential fit.

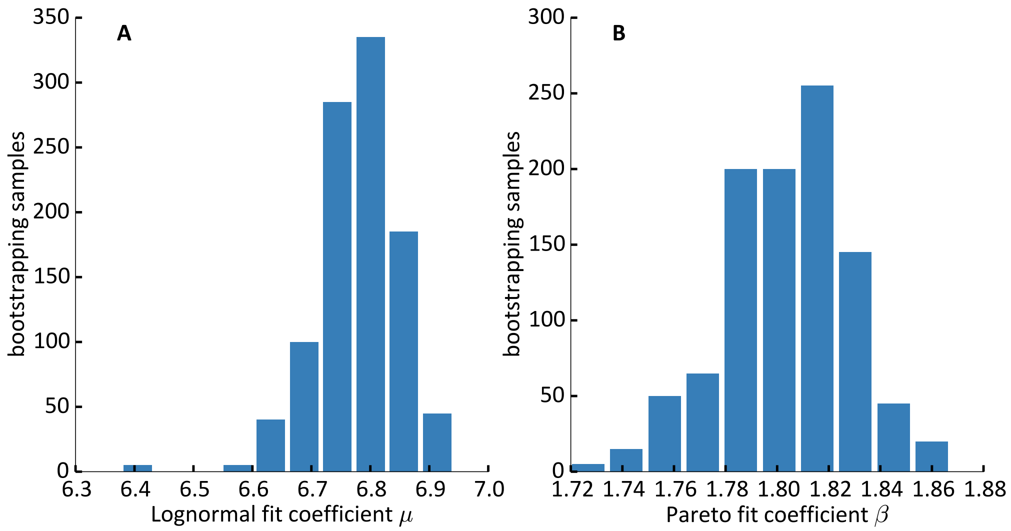

Fig S4. Displacements: distribution of parameters found by bootstrapping. A) The distribution over 1000 bootstrapping samples of the log-normal fit coefficient μ, characterising the aggregated distribution of displacements. **B)**The distribution over 1000 bootstrapping samples of the Pareto fit coefficient β, characterising the tail of the aggregated distribution of displacements. Samples include 100 randomly selected individuals.

of the model. We have verified that the values recovered by the fitting algorithm are consistent with those expected.

We report in table S4 the values of the shift s and scale parameters x_0. The results presented in the main text do not change when we set $x_0 = 0$ except for the distribution of waiting times, where we find Pareto as the best distribution if $x_0 = 0$.

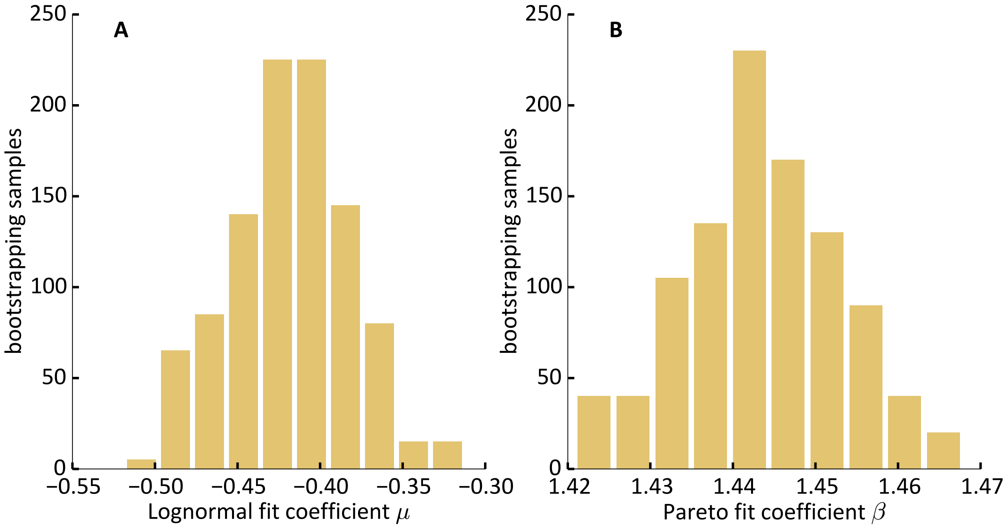

Fig S5. Waiting Times: distribution of parameters found by bootstrapping. **A)**The distribution over 1000 bootstrapping samples of the log-normal fit coefficient μ, characterising the aggregated distribution of waiting times. **B)**The distribution over 1000 bootstrapping samples of the Pareto fit coefficient β, characterising the tail of the aggregated distribution of waiting times. Samples include 100 randomly selected individuals.

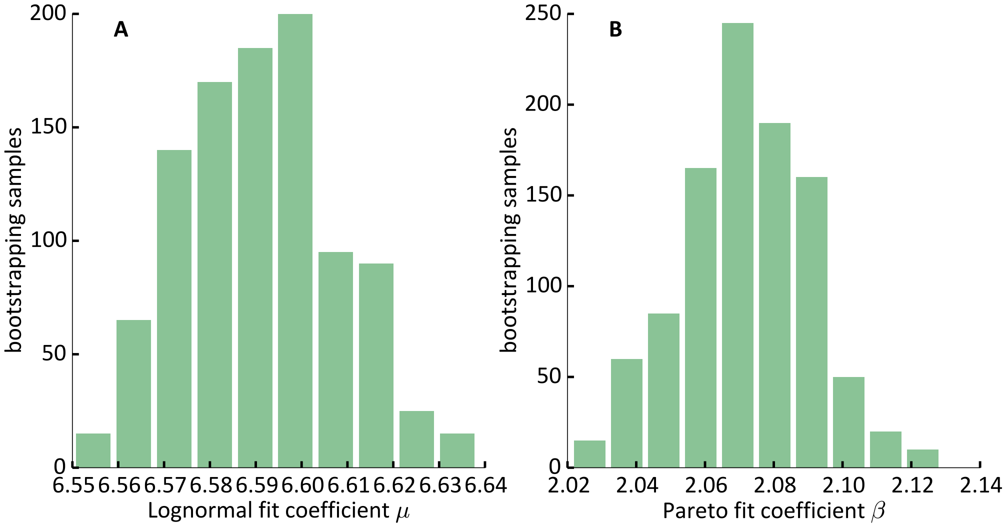

Fig S6. Displacements between discoveries: distribution of parameters found by bootstrapping. **A)**The distribution over 1000 bootstrapping samples of the log-normal fit coefficient μ, characterising the aggregated distribution of displacements between discoveries. **B)**The distribution over 1000 bootstrapping samples of the Pareto fit coefficient β, characterising the tail of the aggregated distribution of displacements between discoveries. Samples include 100 randomly selected individuals.

Further analysis: Selection of the best model among 68 distributions

In the case of the distribution of waiting times, the best model among 68 distributions is the gamma distribution. Results of the gamma fit are shown in figure S11.

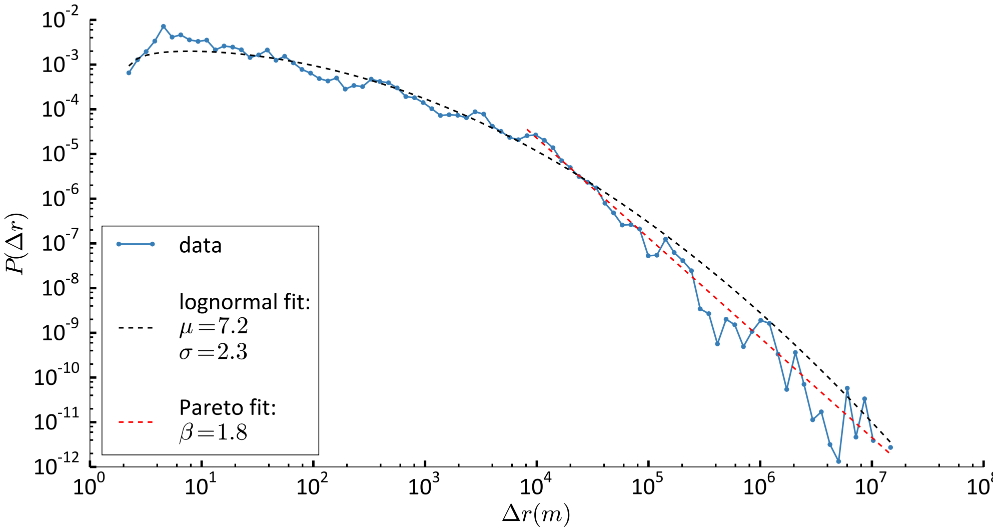

Fig S7. Distribution of displacements for pausing P=15 minutes. Blue dotted line: data. Black dashed line: Log-normal fit with characteristic parameter μ and σ. Red dashed line: Pareto fit with characteristic parameter β for $\Delta r > 7420$ m.

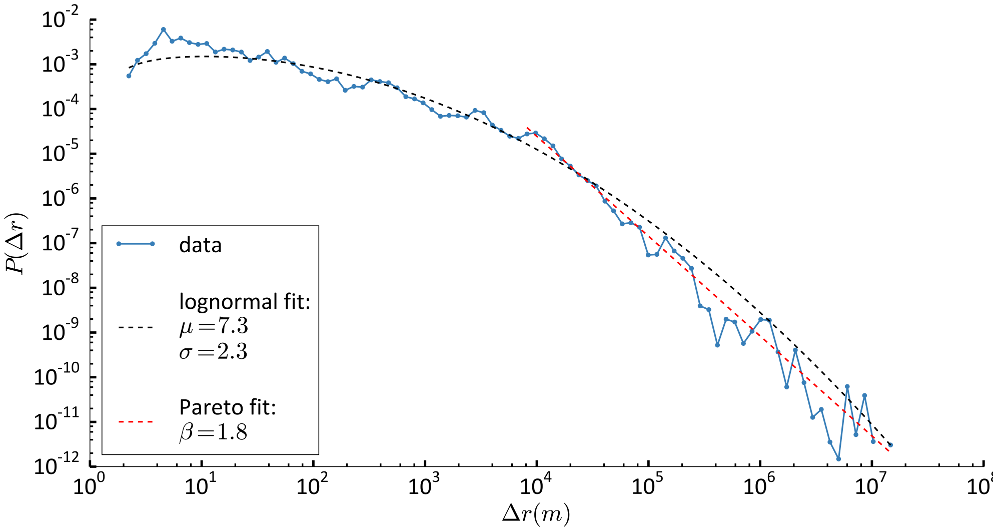

Fig S8. Distribution of displacements for pausing P=20 minutes. Blue dotted line: data. Black dashed line: Log-normal fit with characteristic parameter μ and σ. Red dashed line: Pareto fit with characteristic parameter β for $\Delta r > 7420$ m.

	Shift (Lognormal)	Shift (Pareto)	Scale (Pareto)
Displacements	2.02 m	-11.41 m	7431.83 m
Waiting times	0.18 h	-0.03 h	13.03 h
Discoveries	1.9 m	-1.34 m	2801.35 m

Table S4. The scale and shift parameters. The values of the shift parameter of the Lognormal fit (first column), the shift and scale parameter of the Pareto fit of the distributions' tails (second and third columns).

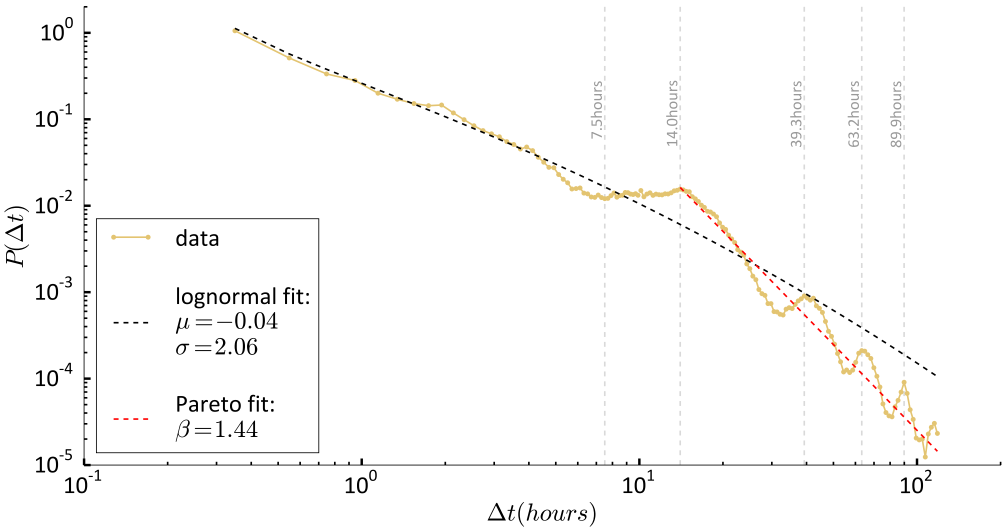

Fig S9. Distribution of waiting times for pausing P=15 minutes. Yellow dotted line: data. Black dashed line: Log-normal fit with characteristic parameter μ and σ. Red dashed line: Pareto fit with characteristic parameter β for $\Delta t > 13h$.

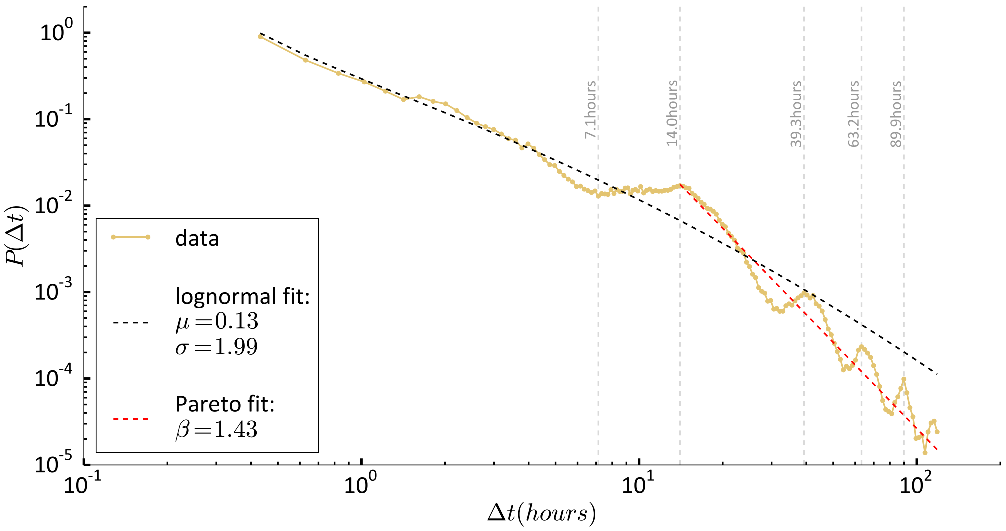

Fig S10. Distribution of displacements for pausing P=20 minutes. Yellow dotted line: data. Black dashed line: Log-normal fit with characteristic parameter μ and σ. Red dashed line: Pareto Fit with characteristic parameter β for $\Delta t > 13$ h.

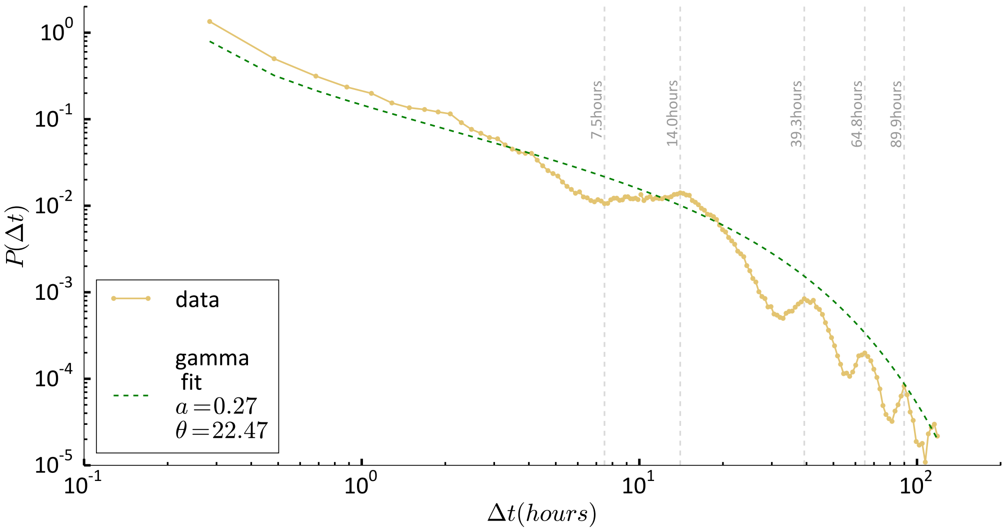

Fig S11. Distribution of waiting times: selection of the best model among 68 distributions. Yellow dotted line: data. Green dashed line: Gamma Distribution fit with characteristic parameters $a = 0.27$ and $\theta = 22.47$

Distributions

The list of distributions is based on the scipy.stats Python [2] module which contains the implementation of over 80 probability distributions, including those reported in the literature on human mobility. We have excluded distributions with more than 3 parameters (including scale and shift), unless they were found in previous studies on human mobility. The distribution considered are the following:
Levy alpha-stable, Anglit, arcsine, Bradford, Cauchy, chi, chi-squared, cosine, double gamma, double Weibull, exponential, exponential power, fatigue-life, Fisk, folded Cauchy, folded normal, Frechet left, Frechet right, gamma, generalized extreme value, generalized Gamma, generalized half-logistic, generalized logistic, Generalized Pareto, Gilbrat, Gompertz, left-skewed Gumbel, right-skewed Gumbel, half-Cauchy, half-logistic, half-normal, hyperbolic secant, inverted gamma, inverse Gaussian, inverted Weibull, General Kolmogorov-Smirnov, Laplace, Levy, left-skewed Levy, log gamma, logistic, log-Laplace, lognormal, Lomax, Maxwell, Nakagami, normal, Pareto, Pearson type III, power-function, power log-normal , power normal, Rayleigh, R, Reciprocal inverse Gauss, Rice, semicircular, Student's T, triangular, truncated exponential, truncated normal, Tukey-Lambda, Truncated Pareto, Uniform, Von Mises, Wald, Weibull maximum, Weibull minimum, wrapped Cauchy

References

1. Sapiezynski P, Gatej R, Mislove A, Lehmann S. Opportunities and Challenges in Crowdsourced Wardriving. In: Proceedings of the 2015 ACM Conference on Internet Measurement Conference. ACM; 2015. p. 267–273.

2. Jones E, Oliphant T, Peterson P, et al.. SciPy: Open source scientific tools for Python; 2001–. Available from: http://www.scipy.org/.
